# Invasive ductal carcinoma within fibroadenoma: a case report

**DOI:** 10.1186/1757-1626-2-174

**Published:** 2009-10-29

**Authors:** Lumturije H Gashi-Luci, Rinë A Limani, Fisnik I Kurshumliu

**Affiliations:** 1 Institute of Pathology, Faculty of Medicine, University Clinical Center of Kosova, Prishtina 10000, Republic of Kosova

## Abstract

**Introduction:**

Fibroadenoma is the most common benign tumor of the female breast with the highest incidence before age 30. Fibroadenoma may be associated with fibrocystic changes, proliferative epithelial changes, and extremely rarely, with non-invasive and invasive cancer.

**Case presentation:**

We present a rare case of a 39 years old female with invasive ductal carcinoma arising within fibroadenoma.

**Conclusion:**

There is a low percentage of fibroadenomas harboring carcinoma; however, all breast lumps should be seriously managed; extirpation and histological examination is recommended.

## Introduction

Fibroadenoma as the most common benign tumor of the female breast in women before age 30 can be accompanied with fibrocystic changes (apocrine metaplasia, cysts and papillary apocrine changes), adenosis, calcifications, and proliferative epithelial changes such as mild, moderate, florid and atypical ductal and lobular hyperplasia. Rarely, lobular and ductal non-invasive and invasive carcinoma may occur within fibroadenoma.

Malignant changes within fibroadenoma are usually an incidental finding following the excision of fibroadenoma.

In 1931 Cheatle and Cutler [1] were the first to describe a carcinoma arising in fiboradenoma.

Azzopardi [2] defined carcinomas involving fibroadenoma as: arising in the adjacent breast tissue engulfing and infiltrating fibroadenoma; in the crevices of a fibroadenoma as well as in the adjacent breast tissue; and carcinoma restricted entirely or at least dominantly to a fibroadenoma.

## Case presentation

39 years old female presented to the doctor with a well defined lump in the upper lateral quadrant of the right breast, of two months duration. Any family history of breast malignancies was denied. Axillary lymph nodes were not palpable.

**Figure 1:**
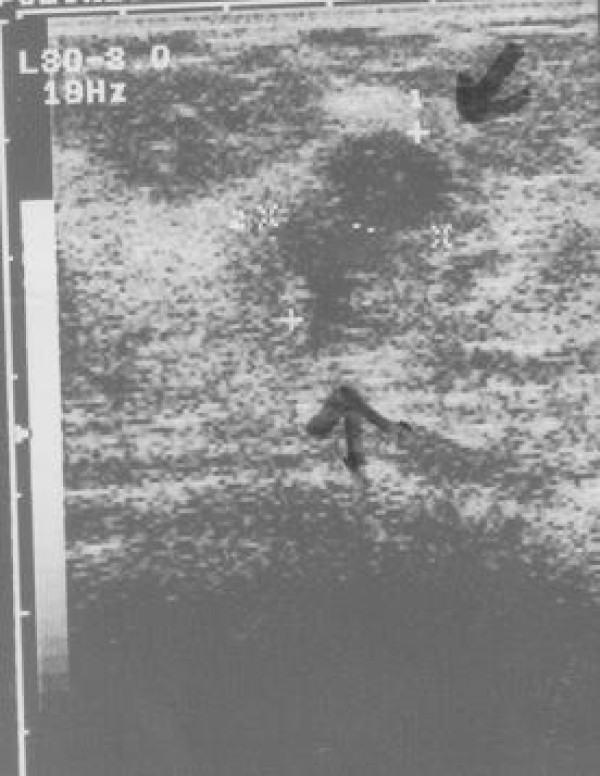
**Ultrasonography**. Partially well defined mass consisted of heterogenous hypoechoic foci.

Ultrasonography revealed a partially well circumscribed heterogeneous hypoechoic mass measuring 25 × 16 mm (Figure 1).

**Figure 2:**
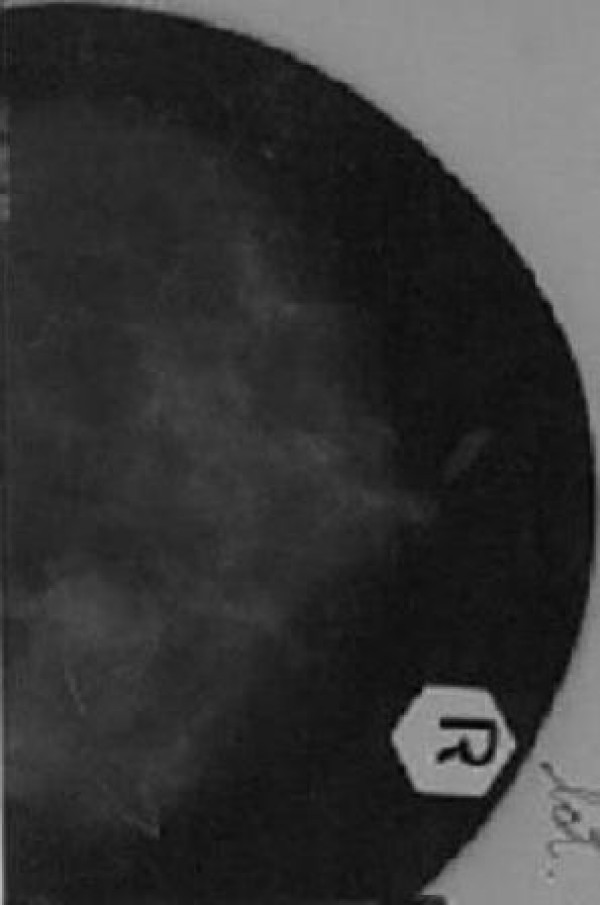
**Mammography**. Solitary lesion with variable density.

In mammography the lesion was partially well circumscribed, solitary and of variable density (Figure 2).

Fine needle aspiration cytology was performed and benign epithelial cells were described in cytology.

Chest X Ray, abdominal ultrasound and all laboratory findings were unremarkable.

Lumpectomy was indicated, but during the procedure the operating surgeon opted for a wide margin of clearance.

### Gross description

Breast tissue specimen measuring 9 × 9 × 4 cm; partially covered with skin measuring 3 × 1 cm. The cut surface revealed a tumor mass measuring 2.6 cm in greatest diameter. Tumor was partially confined by fibrotic capsule but partially infiltrated the surrounding tissue. The tumor tissue was of gray-white color and of moderately increased consistency.

### Histopathology

**Figure 3:**
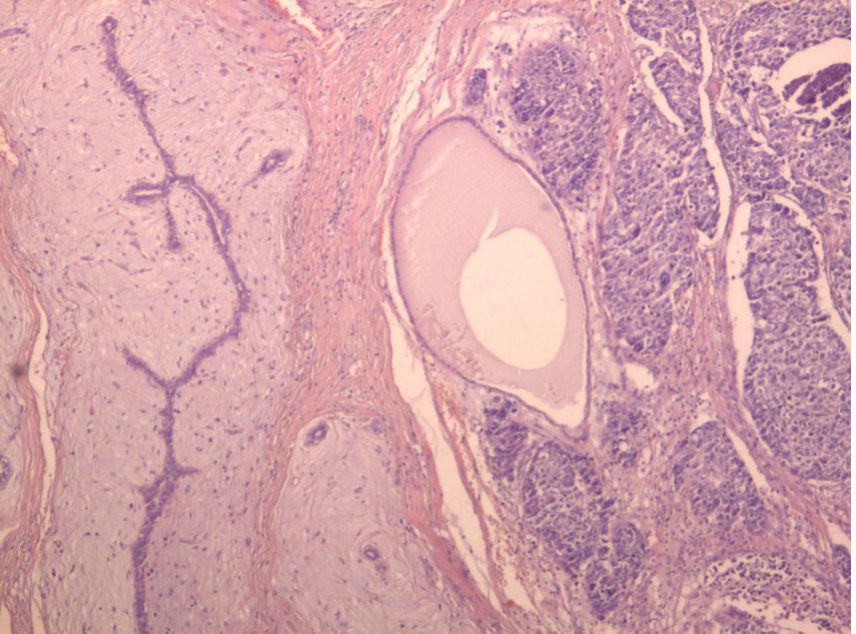
**Ductal carcinoma in situ with focal comedo necrosis and fibroadenoma**. Fibroadenoma In the left hand of the field (×10; H&E stain).

**Figure 4:**
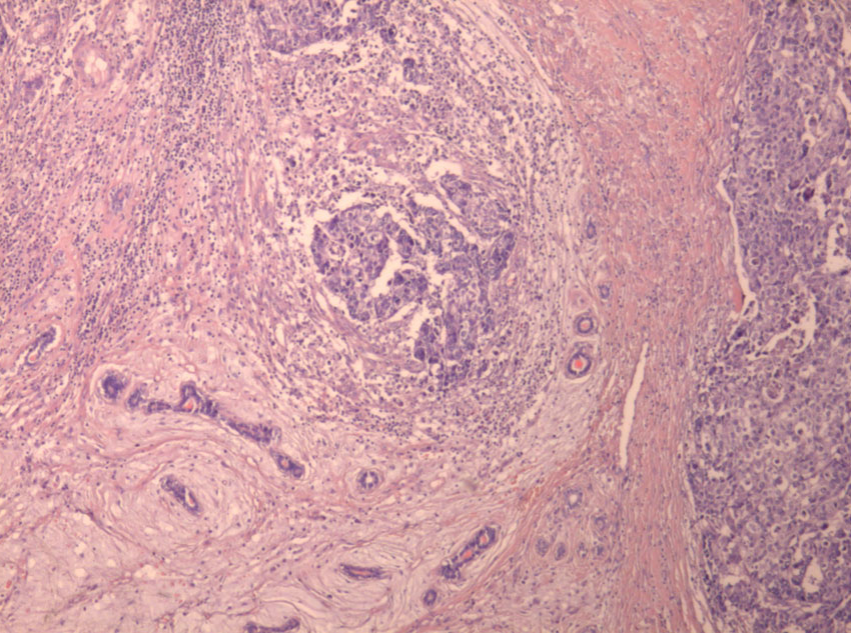
**Infiltrating islands of malignant ductal epithelial cells and fibroadenoma**. Fibroadenoma in the lower left corner of the field (×10 H&E stain).

**Figure 5:**
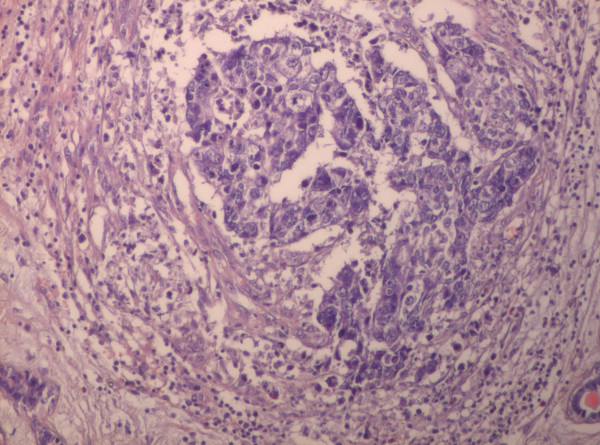
**Infiltrative ductal carcinoma with inflammatory cells in the stroma (×20; H&E stain)**.

Microscopy revealed the tumor to be a fibroadenoma (peri and intracanalicular) with many foci of atypical epithelial changes ranging from atypical ductal hyperplasia to ductal carcinoma in situ (DCIS), mainly of comedo type. The comedo component comprised less then 10% of the tumor mass (Figure 3). Besides the *in situ *component there were foci of infiltrating islands of malignant ductal epithelial cells in the surrounding stroma (Figure 4). Stroma of fibroadenoma and carcinoma was of similar histology with oedema and foci of myxoid change. Inflammatory response around the DCIS and invasive carcinoma (Figure 5) was conspicuous.

The covering skin was unremarkable.

Radical mastectomy was performed subsequently and no residual tumour was found. Sclerosing adenosis was observed in the adjacent breast tissue.

In the axillary dissection specimen there were found 11 lymph nodes measuring 0.2 to 1.1 cm. No metastatic tumor was encountered.

Histology of lymph nodes revealed chronic inflammatory changes with Sinus hystocitosis.

ER, PgR and HER-2 were assessed. The tumor cells did not show any reactivity for ER and PgR hormonal receptors. HER-2 was equivocal (++).

Five months following surgery the patient developed a locally recurring tumor mass measuring 2 cm in greatest diameter. Histology revealed an invasive ductal carcinoma.

## Discussion

The incidence of carcinoma within fibroadenoma is reported to be between 0.1% and 0.3% in a screened population, with a peak age of occurrence between 42 and 44 years [3-8]. Two-thirds of carcinomas within fibroadenoma are lobular and one-third is ductal or mixed ductal and lobular; lobular carcinoma in situ (LCIS) and DCIS have an approximately equal frequency [6-14].

Complex fibroadenomas and proliferative diseases adjacent to fibroadenoma are associated with a slight increase in the risk of breast cancer [6,14].

In an ongoing study at our Institute, we have found hyperplasia in 3.8% among 250 screened cases of fibroadenoma.

The biological behavior of carcinoma occurring within fibroadenoma does not differ from that of breast carcinoma not related to fibroadenoma [5,7,8].

Preoperative clinical criteria and radiological criteria are usually insufficient to suggest that malignant change has occurred in a fibroadenoma, especially in cases with in situ carcinoma.

Because of the heterogeneity of the lesion, FNAC sample is likely to be insufficient for accurate diagnosis, as shown in our case. Hence, the diagnosis of carcinoma arising within fibroadenoma in most instances is made only following excision and histopathological examination of the tumor.

## Conclusion

Despite the low percentage of carcinoma occurring within fibroadenoma we consider that each lump should be seriously managed; extirpation and histological examination is recommended.

FNAC is not always a reliable diagnostic tool.

Special caution has to be taken in females older than 35 years presenting with a fibroadenoma.

## Abbreviations

LCIS: Lobular Carcinoma In Situ; DCIS: Ductal Carcinoma in Situ; FNAC: Fine Needle Aspiration Cytology; USG: Ultrasonography.

## Consent

Written informed consent was obtained from the patient for publication of this case report and accompanying images. A copy of the written consent is available for review by the Editor-in-Chief of this journal.

## Competing interests

The authors declare that they have no competing interests.

## Authors' contributions

LGL has performed the histological examination and was a major contributor in writing the manuscript. RL and FK were major contributors in designing and writing the manuscript. All authors read and approved the final manuscript.
